# Cytotoxic and Genotoxic Effects of Acephate on Human Sperm

**DOI:** 10.1155/2017/3874817

**Published:** 2017-03-14

**Authors:** M. A. Thamali Dhanushka, L. Dinithi. C. Peiris

**Affiliations:** Department of Zoology, Center for Biotechnology, University of Sri Jayewardenepura, Nugegoda, Sri Lanka

## Abstract

Extensive use of organophosphorus pesticides (OPs) could alter semen quality and sperm DNA at different stages of spermatogenesis. Acephate is a highly toxic extensively used OP and, therefore, we aimed to evaluate the effects of acephate on human semen quality and sperm DNA integrity. Sperm collected from healthy males were exposed to 0, 50, 100, and 200 *μ*g/mL of acephate and incubated for 1 h, 2 h, and 3 h. Subsequently, sperm motility, vitality, functional integrity of plasma membrane, sperm capacitation, and DNA damage were examined. Result showed a significant decline of the motility at 100 *μ*g/mL after 3 h and with 200 *μ*g/mL after 1 h, 2 h, and 3 h. Viability was significantly reduced at 200 *μ*g/mL after 2 h and 3 h. Functional integrity was significantly affected at 100 *μ*g/mL after 3h and in 200 *μ*g/mL dose after 2 h and 3h. Similarly, sperm capacitation was significantly affected at 200 *μ*g/mL after 1 h, 2 h, and 3 h and at 100 *μ*g/mL at 3 h. DNA damage was significantly increased only in 200 *μ*g/mL dose after 3 h. The study suggests that exposure to acephate may result in alterations of sperm structure and function thus contributing towards deteriorating in human semen quality triggering infertility.

## 1. Introduction

Over the past few decades several studies have shown destructive reproductive function in animals [1, 2] and in humans [3] owing to man-made chemicals and other toxicants [4]. Unfortunately, relatively few studies have systematically addressed the impact of environmental exposure on human reproductive health. According to recent studies, globally every year couples suffering from infertility (failing to conceive after 12 months of regular unprotected sexual intercourse) accounts for about 60–80 million [3–5]. Ninety-eight percent of male subfertility cases can be attributed mainly to deficient spermatozoa quality [6]. However, subfertility can be regarded as idiopathic and could be a result of risk factors such as environmental endocrine disruptors including pesticides.

Organophosphate pesticides are esters of phosphoric and thiophosphoric acids and their toxicity has been related to their ability to inhibit action of acetylcholinesterase leading to accumulation of acetylcholine at nerve junctions [7]. Many epidemiological studies have recognized the association between exposure to organophosphate pesticides (OPs) and reproductive disorders such as infertility, birth defect, adverse pregnancy outcomes, and perinatal deaths [8]. Organophosphate pesticides are suspected to alter reproductive function by reducing brain acetylcholinesterase activity affecting the pituitary gonadotropin causing infertility [9]. Effects on semen quality could be assessed by parameters such as sperm concentration, percent motile sperm, and percent sperm with normal morphology, along with sperm motion parameters. Several studies have revealed that men exposed to OP had high risk of developing abnormal semen parameters including decreased sperm concentration, decreased sperm motility, decreased sperm count, and higher sex chromosome aneuploidy in sperm [10]. A study conducted in China with different OP showed a clear relationship between presence of urinary insecticide metabolites and sperm concentration and sperm motility in newly married men [11]. In vitro studies carried out using OP have provided evidence that these pesticides could damage DNA in human sperm thus causing genotoxic effects [10].

Among widely used and toxic OPs in Sri Lanka, acephate (*O,S*-dimethyl acetylphosphoramidothioate) is an insecticide, used in agriculture and domestic purposes due to its wide spectrum insecticide property. Acephate is a systemic insecticide with contact and stomach action. The toxic mechanism of acephate is attributed not only to acetylcholinesterase (AchE) inhibition but also to acting as a delayed neurotoxic agent [11]. Sing and Jiang [12] reported that acephate is a potent neurotoxic, mutagenic, carcinogenic, and cytotoxic compound. Similarly, Farag et al. [13] revealed that 14 and 28 mg/kg/day doses of acephate decreased sperm motility and sperm count in adult male mice. Another two studies have confirmed that acephate significantly decrease fertility, sperm dynamics, weight of sex organs, and sex hormones in male albino rats [14]. Therefore, similar to other OPs, there is a potential risk of this compound on reproductive health of humans.

Acephate is extensively used by agriculturalists in Sri Lanka, who are in their prime reproductive age. In the last two to three decades, it has become clear that occupational exposure to pesticides could affect the fertility of men [3], thus indicating high exposure risks of these young growers. The regulation of pesticides is mainly based on animal models and data on human exposure and semen quality continue to be sparse and limited. Therefore, the present study was conducted to investigate the effects of acephate on human male fertility and DNA integrity of sperm cells in vitro.

## 2. Materials and Methods

### 2.1. Test Material

Unformulated acephate (trade name: Surrender; molecular formula: C_4_H_10_NO_3_PS; molecular weight: 183.16 g/mol; purity: 99.0%; recommended field dosage by the manufacturer: 1 g of acephate into 1 L of water) was obtained from Hayleys Agriculture Ltd., Colombo, Sri Lanka. Since acephate is readily soluble in Biggers Whitten Whittingham (BWW), it was used as the control.

### 2.2. Semen Collection and Preparation

Semen samples were collected from healthy male (age 20–30 years) donors from University of Sri Jayewardenepura, Sri Lanka, in a sterile specimen vial. Prior to semen collection donors were given an information sheet and a consent form seeking their willingness to participate in the study. All participating subjects were asked to abstain from any sexual activity for 3 to 5 days before semen collection. Men (mean age of 25.7 ± 5.8) with normal sperm concentration, motility, morphology, viscosity, liquefaction time, and pH and absence of immature forms and leukocytes were selected for the study. Sperm parameters (motility, vitality, hypoosmotic swelling test, and morphology) were evaluated in accordance with World Health Organization guidelines [15]. Since effects of acephate could magnify with abnormal sperm, it is important to select donors with acceptable sperm quality for the study.

Ethical approval (Ethical clearance ref. nnumber: 712/13) was obtained by Ethics Review Committee, Faculty of Medical Sciences, University of Sri Jayewardenepura, Nugegoda, Sri Lanka.

### 2.3. Determination of Dose Levels

To determine the dose levels, LD_50_ value of acephate for sperm motility was carried out. Hence, a sample with 1 mg of acephate in 1 mL of BWW, which is equivalent to recommended field dosage (1 g of acephate into 1 L of water) of acephate, was prepared. The prepared samples were subsequently diluted with BWW to obtain different concentrations of acephate. Sperm suspensions (fixed to 50 × 10^6^ spermatozoa/mL) were placed in Eppendorf tubes and desired volume of either BWW or test solution was added (final volume of the tube is equal to 1 mL). Samples were thoroughly mixd, and were then incubated for 1 h in a humidified incubator (Sanyo Electric Co. Ltd., Tokyo, Japan) at 37°C in 5% CO_2_. After incubation, percentage motility of spermatozoa [15] was determined at each concentration by two independent observers under Olympus microscope with phase-contrast optics (X 400; Olympus Corporation, Japan). Based on this pilot survey to determine indications of toxic doses for semen samples, it was revealed that LD_50_ value for acephate is 200 *μ*g/mL. Hence sublethal concentrations below LD_50_ including LD_50_ value were used in the present study.

Given below is the selected dose levels for the present study:

  High dose: 200 *μ*g/mL (equivalent to LD_50_ value).
  Mid dose: 100 *μ*g/mL.
  Low dose: 50 *μ*g/mL.

Further, we decided to extend the time points from 1 h to 2 h and 3 h to determine the prolonged effects of acephate exposure.

### 2.4. Pesticide Incubation

Fresh semen samples (*n* = 9) were diluted with BWW to obtain final sperm concentration of 40 × 10^6^ spermatozoa/mL. Subsequently samples were incubated either with pesticide at concentrations of 50, 100, and 200 *μ*g/mL or with BWW. Incubations were conducted in 1 mL of final volume for 1, 2, and 3 h at 37°C in an incubator (Sanyo Electric Co. Ltd., Tokyo, Japan) in 5% CO_2_ and 95% O_2_ atmosphere.

### 2.5. Determination of Sperm Motility

Total motile spermatozoa [15] were estimated (approximately 100 cells) by two independent observers under phase-contrast optics (X 400; Olympus Corporation, Japan). Percentage forward motility was calculated.

### 2.6. Cytotoxicity Assay

The viability of spermatozoa was assessed using Eosin Y stain technique [15] and dead and live spermatozoa were calculated. Percentage viability = (total number of cells − number stained/total number of cells) × 100. Total number of cells = 100.

### 2.7. Determination of the Functional Integrity of Sperm Plasma Membrane

The functional integrity of sperm plasma membrane was assessed using the Hypoosmotic Swelling (HOS) test described by Jeyendran et al. [16]. At least 100 spermatozoa were counted per preparation.

### 2.8. Determination of Capacitation

An aliquot of 150 *μ*L of BWW medium was placed in a prewarmed culture dish (35 × 10 mm, Corning, New York, USA) and covered with a prewarmed 22 × 22 mm coverslip. An aliquot of 20 *μ*L sperm sample was added to a corner of the coverslip. The culture dish was placed in an inverted microscope (Diaphot, Nikon, London, UK) fitted with a thermostatically controlled air heated cabinet (Nikon, London, UK) equilibrated at 37°C. Sperm capacitation status was assessed based on hyperactivation by the objective photographic method using an Olympus IX71 phase-contrast microscope. Since hyperactivation is a flagella phenomenon, it is possible to visually assess hyperactivation based on sperm motility; flagella movement patterns were evaluated for the enumeration of nonhyperactivated and hyperactivated sperm [17]. Experiments were performed in duplicate and repeated independently four times.

### 2.9. Determination of DNA Damage in Spermatozoa

Sperm smears were prepared on cleaned microscopic slides and the percentage of sperm with normal DNA was determined by counting 500 sperm under fluorescence microscopy (BH-2, Olympus Ltd., Japan) with excitation of 490 nm. The observation time period for a single field took less than 40–50 sec. The sperm exhibiting yellow to red color was scored as denatured DNA and sperm exhibiting green color was scored as normal DNA [18].

### 2.10. Statistical Analysis

Sperm counts of adult males were normally distributed and were compared between groups with two-way analysis of variance (ANOVA) using Minitab software package (Minitab Co., USA) for the main effect of pesticides. Where a significant treatment effect was found, differences among individual group means were tested by “Tukey 95%.” The data are expressed as mean ± Standard Error Mean (SEM). *P* value was set to *P* ≤ 0.05.

## 3. Results

### 3.1. Sperm Motility

When compared with control, percentage motility was significantly (*P* ≤ 0.05) decreased (Table 1) at mid dose (100 *μ*g/mL) by 16% after 3 h of incubation. In contrast, highly significant (*P* ≤ 0.001) reduction was recorded in 200 *μ*g/mL after 1 h, 2 h, and 3 h of incubation when compared to controls. Further, reduction of motility is clear in each dosage with increasing incubation time.

### 3.2. Cytotoxicity

Percentage viability was decreased (Table 1) in all treatment groups with time, but significant reduction (by 20%; *P* ≤ 0.05) was recorded in 200 *μ*g/mL (high dose) after 2 h after incubation when compared to controls. Highly significant (*P* ≤ 0.001) reduction was recorded in high dose after 3 h of incubation and it was decreased by 31% compared to the control.

### 3.3. Functional Integrity of Sperm Plasma Membrane

Table 1 displays the results of functional integrity of sperm cells after 1 h, 2 h, and 3 h of incubation. Significant reductions (*P* ≤ 0.05) of functional integrity were recorded in mid dose by 13.5% after 3 h and also in high dosage by 26% after 2 h of incubation when compared with their controls. Highly significant (*P* ≤ 0.001) reduction was recorded in high dosage (by 35%) after 3 h of incubation.

### 3.4. Sperm Capacitation

Reduction in capacitated spermatozoa was observed in all treatment groups (Table 1). The percentage of capacitated spermatozoa was significantly reduced (*P* ≤ 0.05) in 100 *μ*g/mL dose by 18% after 3 h of incubation and at high dosage by 32%, 36%, and 38%, respectively, after 1 h, 2 h, and 3 h of incubation.

### 3.5. DNA Damage in Spermatozoa

Percentage of DNA damaged spermatozoa count was increased with increasing of dosage and incubation time. But significant (*P* ≤ 0.01) increase of DNA damage was recorded only at high dosage (by 33%) after 3 h of incubation. Results are summarized in Table 1.

## 4. Discussion

OP insecticides are extensively used in Sri Lanka with little or no protection by the users and individuals are thus at high exposure risk. Moreover, these agriculturalists in their prime reproductive ages are inevitably exposed over a long period of time. The present study was designed to demonstrate the association between acephate, an OP with semen quality, and DNA damage in sperm over different incubation time points. Results obtained from the present study indicated that acephate impairs sperm motility, sperm viability, membrane integrity, and sperm capacitation and induced DNA damage in vitro. Similar results were obtained with chlorinated hydrocarbon [19], organophosphates [20, 21], and benzene metabolites [22].

The significant reduction of sperm motility observed in both mid (100 *μ*g/mL) and high (200 *μ*g/mL) doses of acephate could result in impairment of sperm fertilizing ability [23]. After 3 h exposure to the highest dose, the percentage motility of the spermatozoa was reduced below the values recommended by WHO. Sperm motility is considered to be one of the most sensitive detectors of sperm cytotoxic effects and reduction in sperm fertilizing ability. Changes in mitochondrial membrane potential by different pesticides could lead to reduction in sperm motility [24]. Sperm motility also depend on the intense transformation and energy expense produced via the mitochondria oxidative pathways and pesticides could interfere with oxidative pathways producing delayed sperm motility eventually leading to cell death [25]. Furthermore, pesticide metabolites could interfere with mitochondrial oxidative reactions leading to production of excess reactive oxygen species (ROS) decreasing the production of ATP resulting in motility impairments [25]. Oxidative stress has been reported to be the primary mechanism of organophosphate including acephate [26] toxicity. Loss of integrity can lead to an increase in membrane permeability and loss of capacity to regulate the intracellular concentrations of ions involved in control of sperm movement [27]. The overall effect of membrane damage might be responsible for continuous decrease in sperm motility and viability after ejaculation [25].

The viability is the proportion of live spermatozoa determined by the evaluation of cellular and or membrane integrity [28]. Eosin staining test has provided information on sperm membrane structural integrity, while HOS test has provided information on sperm membrane functional integrity [29]. Viability of spermatozoa is associated with intact, functional, and semipermeable plasma membranes [22]. Pesticides could induce calcium ion rise causing damage to sperm membranes, thus affecting sperm viability long before they reach the oocyte [30]. Changes in plasma membrane observed in the present study could result in reduction in sperm motility, viability, and membrane integrity [22].

Hyperactivation is in an indication of sperm completion of capacitation and is essential for penetration through the cumulus mass and zona pellucida [4]. In the present study it was shown that hyperactivated spermatozoa decreased in high concentration and decreased with increased incubation time, indicating impaired sperm capacitation. Sperm membrane and mitochondria are essential in powering hyperactivation and any damage to sperm membrane and sperm mitochondria may lead to reduction in capacitated sperm [25]. OP is known to impair capacitation through inhibition of AchE when incubated with sperm in vitro [31]. Nevertheless, upon addition of sufficient amounts of AchE or AchE mimic enzymes to the medium, the effect was reversed [22], thus indicating importance of activity of AchE of the sperm membrane to initiate capacitation.

The present study reveals a significant damage to double-stranded DNA at highest dosage (200 *μ*g/mL) after 3 h of incubation. Integrity of sperm DNA is vital to transmit genetic information during reproduction and any damage to DNA could result in infertility [25]. DNA bases and phosphodiester backbones are particularly susceptible to oxidative stress induced damage because their plasma membranes contain large quantities of polyunsaturated fatty acids and their cytoplasm contains low concentrations of scavenging enzymes [25]. Hence, high levels of ROS could result in double-strand DNA breaks commonly observed in the spermatozoa of infertile men [32]. It has been recorded that acephate significantly increases the cellular reactive oxygen species (ROS) resulting in modifications of DNA by forming base pair errors and strand breaks [33]. DNA fragmented spermatozoa are less motile and less susceptible to hypoosmotic swelling indicating lower functional integrity of sperm membrane thus leading to reduction in sperm function with increasing DNA damage [34]. Pesticides inducing DNA damage in leukocytes could also lead to DNA damage in spermatozoa [35].

## 5. Conclusions

The present study is suggestive of possible impairment of sperm function with acephate exposure. High concentration of acephate (200 *μ*g/mL, which is equivalent to LD_50_) tested in this study altered human sperm motility, viability, functional integrity of plasma membrane, and sperm capacitation and induced DNA damage in vitro. After considering the results and natural human biological responses such as degradation and clearance of chemicals, it could be recommended that tested higher dosage (200 *μ*g/mL) and above may cause a risk for human health.

## Figures and Tables

**Table 1:** 

Parameters	Time of exposure (h)	Treatment (*µ*g/mL)			
		0	50	100	200
Motility (%)	1	86.5 ± 2.4	82.9 ± 1.5	75.7 ± 2.7	62.0 ± 0.9^*∗*^
	2	79.3 ± 2.1	76.3 ± 1.9	74.1 ± 3.1	58.9 ± 2.8^*∗∗*^
	3	72.3 ± 1.9	67.3 ± 2.5	60.2 ± 2.6^*∗*^	37.2 ± 4.8^*∗∗*^

Viability (%)	1	88.4 ± 1.6	84.4 ± 1.2	80.4 ± 1.3	74.4 ± 1.8
	2	78.8 ± 1.0	76.5 ± 1.2	74.9 ± 1.3	62.9 ± 1.6^*∗*^
	3	70.8 ± 0.5	68.4 ± 1.6	66.8 ± 1.1	47.8 ± 1.4^*∗∗*^

Sperm swelling (%)	1	81.2 ± 5.3	78.9 ± 3.9	72.7 ± 2.6	65.4 ± 3.7
	2	76.6 ± 3.8	73.8 ± 2,9	68.8 ± 2.7	56.6 ± 2.9^*∗*^
	3	69.7 ± 2.9	69.2 ± 3.2	60.2 ± 3.1^*∗*^	45.2 ± 2.9^*∗∗*^

Capacitated spermatozoa (%)	1	38.5 ± 1.6	38.5 ± 1.4	36.1 ± 2.6	25.7 ± 2.7^*∗*^
	2	35.7 ± 2.1	33.0 ± 3.8	34.5 ± 2.6	22.5 ± 2.6^*∗*^
	3	33.3 ± 1.9	32.3 ± 2.6	27.3 ± 3.1^*∗*^	20.7 ± 2.9^*∗*^

DNA damage (%)	1	28.7 ± 3.1	28.3 ± 3.4	28.1 ± 2.6	30.7 ± 3.5
	2	27.3 ± 3.2	28.0 ± 2.8	28.3 ± 2.6	30.4 ± 2.7
	3	30.1 ± 3.9	33.1 ± 2.8	34.1 ± 1.9	40.0 ± 3.4^*∗∗*^

Values represent mean ± SEM (*n* = 9). ^*∗*^*P* ≤ 0.05. ^*∗∗*^*P* ≤ 0.001.

## Abbreviations

BWW: Biggers Whitten Whittingham
HOS: Hypoosmotic solution
NaCl: Sodium chloride
OP: Organophosphate pesticide
ROS: Reactive oxygen species.
